# Plasma-derived Exosomes Reverse Epithelial-to-Mesenchymal Transition after Photodynamic Therapy of Patients with Head and Neck Cancer

**DOI:** 10.18632/oncoscience.410

**Published:** 2018-04-29

**Authors:** Marie-Nicole Theodoraki, Saigopalakrishna S. Yerneni, Cornelia Brunner, Joannis Theodorakis, Thomas K. Hoffmann, Theresa L. Whiteside

**Affiliations:** ^1^ Department of Pathology, University of Pittsburgh School of Medicine and UPMC Hillman Cancer Center, Pittsburgh, PA 15213, USA; ^2^ Department of Otorhinolaryngology, Head and Neck Surgery, University of Ulm, Germany; ^3^ Department of Biomedical Engineering, College of Engineering, Carnegie Mellon University, Pittsburgh, PA 15217, USA; ^4^ Department of Urology, Geniki Kliniki, Thessaloniki, Greece; ^5^ Departments of Immunology and Otolaryngology, University of Pittsburgh School of Medicine, Pittsburgh, PA 15213, USA

**Keywords:** plasma-derived exosomes, exosome-mediated reprogramming, epithelial-to-mesenchymal transition, head and neck cancer, photodynamic therapy

## Abstract

Photodynamic therapy (PDT) is a palliative treatment option for head and neck squamous cell carcinoma (HNSCC) patients which induces local inflammation and alters tumor cell morphology. We show that exosomes in plasma of HNSCC patients undergoing PDT reprogram tumor cells towards an epithelial phenotype. Nine HNSCC patients were treated with PDT and plasma was collected prior to and at three timepoints after therapy. Exosome levels of E-Cadherin, N-Cadherin and TGF-β1 were tested by flow cytometry. Exosomes were co-incubated with cancer cells, and changes in expression of EMT markers were evaluated as were proliferation, migration, chemotaxis and invasiveness of tumor cells. Exosomes harvested pre- and 24h after PDT were enriched in N-Cadherin and TGF-β1. They induced the mesenchymal phenotype and up-regulated Vimentin and transcripts for Snail, Twist, α-SMA, Slug and ZEB1 in epithelial tumor cells. These exosomes also enhanced tumor proliferation, migration and invasion. In contrast, exosomes obtained on day 7 or 4-6 weeks after PDT carried E-cadherin, restored epithelial morphology and EpCAM expression in tumor cells, down-regulated expression of mesenchymal genes and inhibited proliferation, migration and invasion. The PDT-mediated conversion from the mesenchymal to epithelial tumor phenotype was mediated by exosomes, which also served as non-invasive biomarkers of this transition.

## INTRODUCTION

Despite the currently available treatment options for patients with advanced Head and Neck Squamous Cell Carcinoma (HNSCC), the disease outcome remains poor, mostly due to locoregional tumor recurrence. Therapy options are limited for patients with recurrent HNSCC. The standard of care is palliative chemotherapy [1]. Today, PD-1 inhibitors represent a newly approved therapeutic option for these patients [2, 3]. An alternative treatment option for selected cases of recurrent and therapy- refractive HNSCC is the photodynamic therapy (PDT) with Temoporfin (Foscan®, Biolitec, Austria) [4-7]. Effects of PDT on the tumor may vary. PDT might induce tumor necrosis and tissue damage, leading to local inflammation, infiltration of immune cells and activation of long-term immune responses [8-10]. We have previously shown that PDT has a significant impact on the expression of cancer testis antigens (CTA) in human HNSCCs and on the functionality of immune cell populations in blood samples of patients [11].

The process of epithelial-to-mesenchymal transition (EMT), also referred to as “mesenchymalization,” involves a series of molecular and genetic changes in tumor cells that culminate in a conversion of the epithelial phenotype to a highly aggressive and invasive mesenchymal state [12, 13]. The change in cellular morphology that occurs during this conversion is characterized by a loss of cell adhesion and the acquisition of the migratory, invasive signature that favors cancer [14]. The molecular and genetic species that characterize EMT include transcription factors such as Snail, Slug, ZEB1, Twist and others [15, 16], changes in expression levels of cell surface proteins, such as a loss of E-cadherin and the appearance of N-cadherin, expression of novel micro RNAs and changes in cytoskeletal proteins [13]. Effects of PDT on the EMT progression in various cancer types have been controversial [17-22]. For example, Della Pietra et al. reported that sub-optimal levels of PDT can induce EMT in prostate cancer cells [23], whereas Mao et al. showed that PDT combined with carboplatin suppressed EMT in laryngeal carcinomas [24]. The mechanisms responsible for these effects of PDF on the course of EMT remain unclear.

Tumor-derived exosomes (TEX) are cell derived nano-sized extracellular vesicles that play a crucial role in intercellular communication and in reprogramming of the tumor microenvironment (TME) [25]. We and others have reported that HNSCC are strong TEX producers [26] and that the plasma of patients with HNSCC is highly enriched in TEX [27]. TEX carry numerous immunosuppressive proteins as well as molecules promoting EMT [27-29]. Emerging evidence suggests that TEX can modulate EMT in carcinomas [30]. Franzen et al. [28] showed that urothelial cells treated with exosomes produced by bladder cancer increased expression levels of mesenchymal markers, e.g., alpha-smooth muscle actin (αSMA), while down-regulating expression levels of epithelial markers such as E-cadherin as well as increasing their migration and invasion capabilities. Further, Min et al. reported that the exosome cargo of esophageal carcinoma cell lines was altered following radiation therapy, and that these altered exosomes promoted tumor metastasis by EMT induction [29]. Taken together, current reports indicate that: (a) cancer therapies can influence the morphology and behavior of tumor cells, in some cases promoting rather than inhibiting EMT and (b) tumor-derived exosomes are involved in the regulation of EMT, reflecting the parent cell responses to treatment regiments. However, most of the studies reporting exosome participation in EMT have been performed with cell lines or in mouse models. To the best of our knowledge, there is no study addressing the *in vivo* influence of oncological therapies on the ability of tumor-derived exosomes to modulate EMT in patients undergoing treatments.

This is the biggest cohort of HNSCC patients undergoing PDT with a standardized sample acquisition. Here, we show for the first time that exosomes isolated from plasma of patients with HNSCC treated with and responding to PDT recapitulate molecular characteristics of the parent tumors, serving as markers of PDT-induced molecular changes in the parent tumor. Moreover, these exosomes upon co-incubation with carcinoma cells can reverse the EMT phenotype of recipient cells and suppress their migration as well as invasiveness.

## RESULTS

### Characteristics and molecular content of the plasma-derived exosomes prior to and after PDT

Extracellular vesicles (EVs) isolated by size exclusion chromatography from plasma of HNSCC patients treated with PDT meet the criteria attributed to exosomes (Supplementary Figure 1). They have a size range of 30-150nm based on DLS and NTA analyses. Transmission electron microscopy (TEM) images show their vesicular morphology and confirm the vesicle diameter approaching 100nm. Western blots indicate the presence of Tsg101, an endocytic marker, and CD63, a tetraspanin, in the exosome cargo.

**Figure 1:**
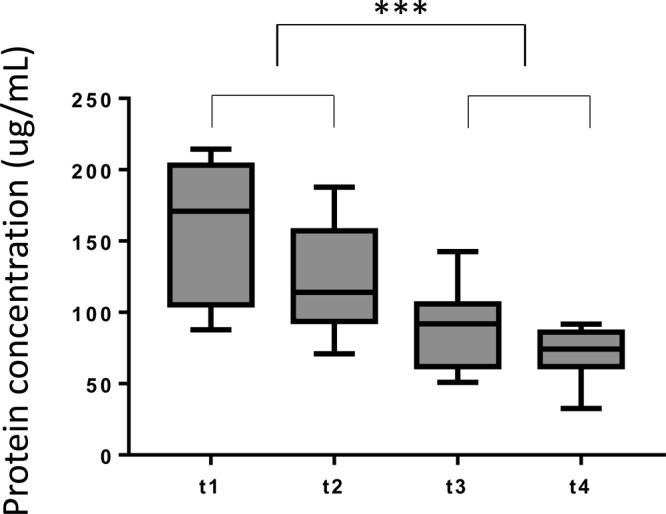
Protein concentrations of plasma-derived total exosome fractions for all HNSCC patients and for all time points (n=9) Exosomes harvested from patients before and immediately after PDT (t1 and t2) have a significantly higher protein concentration than exosomes harvested at the later time points after PDT (t3 and t4). 2-tailed paired t-test, ^***^p<0.0001.

Exosomes were isolated from plasma specimens obtained prior to (t1) and serially at 3 different tine points (t2, t3 and t4) after PDT. The protein content was measured in all exosome fractions. The mean protein level in exosome fractions isolated from pre-therapy plasma specimens (t1) was 161 μg/mL (Figure 1). Following PDT, protein concentrations steadily decreased, reaching the mean level of 72 μg/mL at t4. The decreasing protein levels in serially collected exosome fractions from plasma of patients responding to PDT suggest that the total exosome protein might serve as an indication of response to this therapy in HNSCC patients.

**Figure 2:**
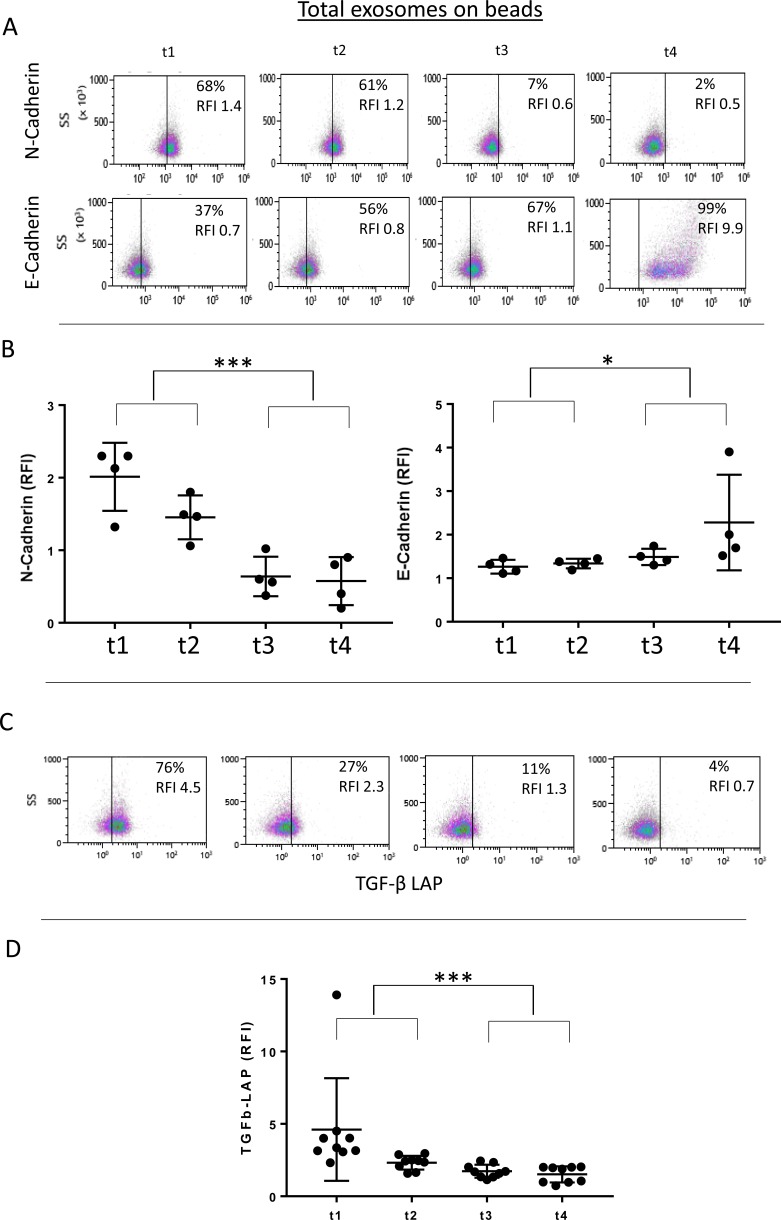
Alterations in the molecular cargo of exosomes harvested from HNSCC patients undergoing PDT (**A**) Representative flow cytometry density plots for E-Cadherin and N-Cadherin levels carried on total exosomes harvested at t1, t2, t3 and t4. Results are presented as RFIs as well as % positive exosomes. (**B**) Combined flow cytometry results are shown for 4 patients. Note the decreasing levels of N-Cadherin during therapy compared to the increasing levels of E-Cadherin. (**C**) Representative and combined (**D**) data for levels of TGF-β1 carried on exosomes harvested from HNSCC patients undergoing PDT (n=9). 2-tailed paired t-test, ^*^p=0.007; ^***^p<0.0002.

The cargos of plasma exosomes obtained prior to and at the specified time points after PDT were evaluated by on-bead flow cytometry using total exosome fractions isolated from plasma. The exosome cargo was found to contain N-Cadherin (Figure 2A and B). The N-Cadherin levels were highest in the pre-therapy (t1) exosomes, and they decreased in exosomes isolated from the patients' plasma after PDT (t3, t4). We also measured levels of E-Cadherin in the exosome cargo and found that following PDT, the E-Cadherin content increased to reach the highest level in exosomes collected at t4, i.e., 4-6 weeks after PDT (Figure 2A and B). The observed decreases in N-Cadherin and corresponding increases in E-Cadherin levels in exosome fractions following PDT suggested that these changes might reflect PDT-induced reversal of EMT occurring in parent tumor cells.

Since it has been reported that TGF-β1 is one of the main promoters of EMT [31], we also investigated levels of TGF-β1 in the cargo of total exosomes obtained from pre- and post PDT plasma of all the patients. We found that exosomes isolated from pre-PDT plasma contained high levels of TGF-β1, and that following PDT, these levels significantly decreased to reach the lowest levels in exosomes harvested at t4 (4-6 weeks after PDT) as shown in Figure 2C and D.

### Exosomes isolated from plasma of HNSCC patients undergoing PDT induce phenotypic changes in recipient cancer cells

**Figure 3:**
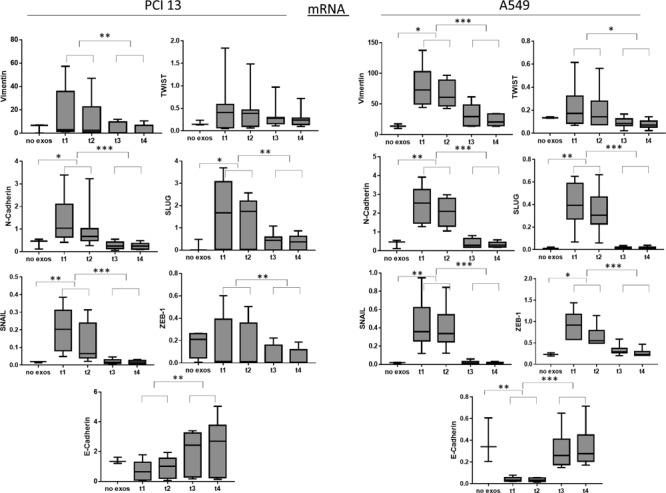
PCR results for mRNA levels in PCI13 and A549 cells co-incubated with plasma exosomes from HNSCC patients undergoing PDT Exosomes harvested at all four time points were harvested and co-incubated with tumor cells (n=9). Following co-incubation, qRT- PCR using mRNA obtained from recipient cells was performed as described in Methods. Results are presented as fold increases using the housekeeping gene, *HPRT1* as a reference gene. Note the increased expression levels of mesenchymal mRNA for Snail, Slug, Twist, Vimentin, N-Cadherin and Zeb1 at t1 and t2. The epithelial marker, E-Cadherin, was significantly increased at t3 and t4. Cancer cells co- incubated with no exosomes were used as negative controls (-). 2-tailed paired t-test, ^*^p<0.05; ^**^p<0.004; ^***^p<0.0001.

Previous studies have demonstrated that high expression levels of mesenchymal markers, such as Vimentin, Snail or Twist, are associated with poor prognosis in HNSCC [32]. To determine whether exosomes obtained from plasma of the HNSCC patients treated with PDT could induce expression of mesenchymal markers in epithelial tumor cells such as PCI13 and A549, co-incubation experiments were next performed. Total exosomes isolated from plasma at different time points pre- and post PDT were co-incubated with tumor cells as described in Methods. Total mRNA was isolated from tumor cells treated with exosomes and qRT–PCR was performed to evaluate gene expression of *Vimentin, N-Cadherin, Snail, Twist, Slug and ZEB-1* in recipient cells. As shown in Figure 3, exosomes isolated from plasma prior to PDT induced the highest expression of these genes in the recipient tumor cells. In contrast, exosomes obtained after PDT, progressively lost the ability to induce transcription of these genes in the recipient cells.

Further, pre-PDT exosomes failed to induce the mRNA transcript for *E-Cadherin* in the same recipient tumor cells. However, exosomes harvested after PDT (on day 7 or week 4-6) significantly up-regulated mRNA transcript for *E-Cadherin*. The latter effect was especially prominent in A549 cells. There was no significant upregulation of *ZEB-1* mRNA in PCI-13 cells co-incubated with exosomes as opposed to significant up-regulation of *ZEB-1* message in A549 cells indicating variability in responses to exosome uptake by different tumor cells (Figure 3).

**Figure 4:**
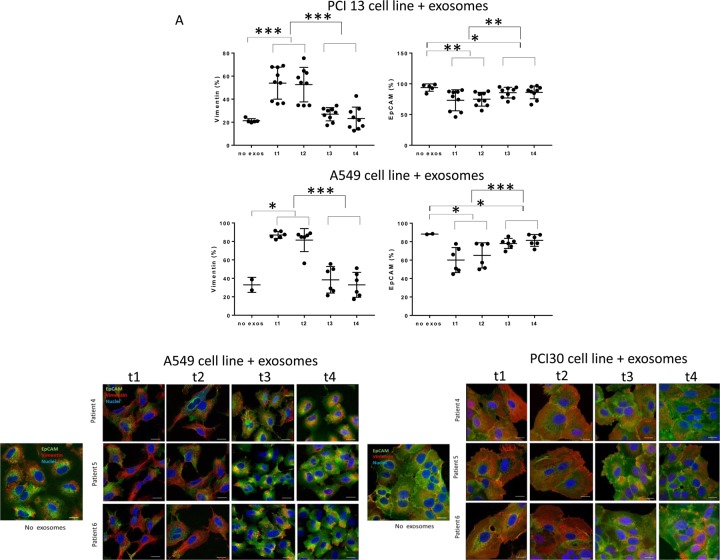
Alterations in expression levels of EpCAM or Vimentin in PCI-13 and A549 cells co-incubated with plasma exosomes from HNSCC patients undergoing PDT Exosomes harvested at all four time points were co-incubated with tumor cells. Staining for EpCAM or Vimentin expression on the recipient cells was performed and cells were examined by flow cytometry in A or by confocal microscopy in B. Note the decreasing levels of Vimentin at t3 and t4 and the simultaneously increasing EpCAM levels. Note also that the morphology of A549 cells appears altered to cuboidal after co-incubation with exosomes obtained at t3 and t4, while treatment with exosomes from t1 and t2 induces a spindle-like morphology (in B). Scale bar = 20µm. 2-tailed paired t-test, ^*^p<0.05; ^**^<0.004; ^***^p<0.0001. Cancer cells incubated with medium alone were used as negative controls (no exos).

To determine whether transcriptional changes induced by exosomes from plasma of HNSCC patients translate to protein expression in PCI-13 and A549 cells, 72h co-incubation with exosomes was followed by immunofluorescence and confocal microscopy (Figure 4A and B). Pre-PDT exosomes completely down-regulated expression of EpCAM and concomitantly up-regulated Vimentin expression in PCI-13 and A549 cells. Exosomes harvested from plasma at t2 (24h after PDT) induced similar effects. In contrast, exosomes obtained at t3 and t4 after PDT induced strong expression of EpCAM and down-regulated Vimentin in both recipient cells (Figure 4).

These experiments show that plasma-derived exosomes of the HNSCC patients undergoing PDT when co-incubated with tumor cells induced transcriptional and translational alterations in the key components of the EMT pathway in the recipient cells. Further, while exosomes isolated prior to or immediately after PDT increased expression of mesenchymal proteins, those harvested from plasma after PDT, at the time corresponding to PDT-induced clinical responses, increased expression of epithelial markers and lost mesenchymal Vimentin.

### Exosomes isolated from plasma of HNSCC patients undergoing PDT induce functional changes in recipient tumor cells

**Figure 5:**
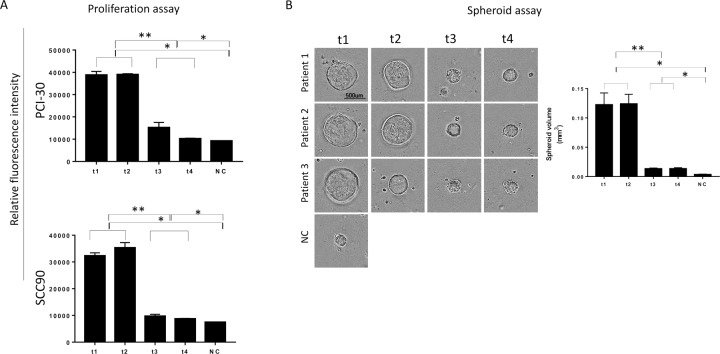
Proliferation of cancer cells co-incubated with plasma exosomes obtained from HNSCC patients undergoing PDT A. Proliferation assay using PCI-30 and SCC90 cells incubated ± exosomes from plasma of HNSCC patients (n=3). Exosomes from all 4 time points were tested. In A, results are presented as relative fluorescence intensity. Proliferation of cancer cells was increased after co-incubation with t1 and t2 exosomes, whereas cells co-incubated with t3 and t4 exosomes behave like the untreated controls. In B, the 3D spheroid assay with PCI-30 cancer cells co-incubated with total exosomes from different time points before and after PDT (n=3). Note the significantly higher spheroid volume after co-incubation with t1 and t2 exosomes. In contrast, t3 and t4 exosomes behaved like negative controls. 2-tailed paired t-test, ^*^p<0.02; ^**^p<0.002.

**Figure 6:**
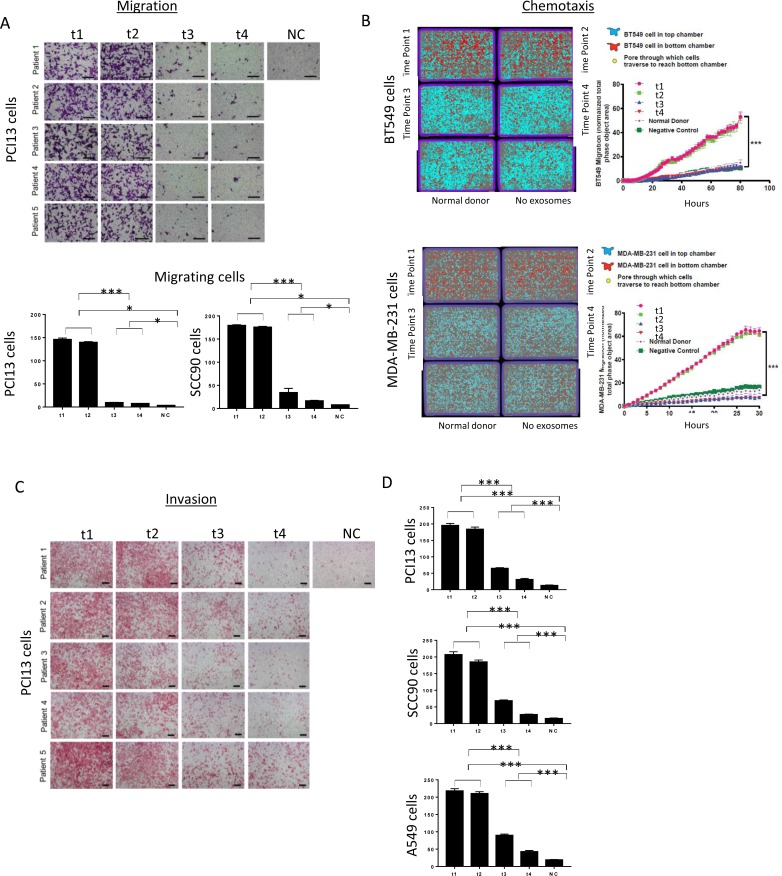
Migration, chemotaxis and invasiveness of tumor cells co-incubated with plasma exosomes obtained from HNSCC patients undergoing PDT In A, representative and combined results of migration of PCI13 cells treated with total exosomes of 5 HNSCC patients. Note that t1 and t2 exosomes increased migration in tumor cells, whereas no migration was observed with tumor cells co-incubated with t3 and t4 exosomes. Scale bar = 50 µm. In B, representative results of the chemotaxis assay using two different cell lines, BT549 and MDA-MB-231 cells. Total exosomes of patient #2 obtained before and after PDT were used as chemoattractant. The data for exosomes of two additional patients are in Suppl. Figure 2. Chemotaxis towards t1 and t2 exosomes is significantly greater than that toward t3 and t4 exosomes. The graphs demonstrate exosome-driven chemotaxis (patients' vs normal controls' exosomes and no exosome controls) of cancer cells over time. In C, representative results of the invasion assay using PCI13 cell line. Invasion of cancer cells co-incubated with t1 and t2 was significantly greater than that with t3 and t4 exosomes. Scale bar = 200 µm. In D, combined results for all three cell lines, PCI13, SCC90 and A549 used in invasion assays performed with pre- and post-PDT exosomes of 5 HNSCC patients. 2-tailed paired t-test and one-way Anova, ^*^p<0.02, ^***^p<0.0007.

EMT has been shown to be associated with cancer invasion and metastasis in several malignancies, including HNSCC [33-35]. We, therefore, expected that plasma exosomes of HNSCC patients undergoing PDT that induced EMT-related phenotypic changes in recipient cells might also alter their functions. Indeed, exosomes harvested from plasma at t1 and t2 strongly promoted proliferation of recipient tumor cells (Figure 5A), while those harvested at t3 and t4 lost the ability to promote tumor growth. Similarly, spheroid formation by tumor cells was augmented by the pre-PDT and 24h post-PDT exosomes but was no longer supported by exosomes collected at t3 and t4 (Figure 5B). The data suggest that tumor cell growth is only promoted by exosomes produced when the tumor has the mesenchymal phenotype, and it ceases to be promoted with cell conversion to the epithelial phenotype. Not only tumor cell proliferation but also motility and chemotaxis of tumor cells are differentially modulated by exosomes from plasma of HNSCC patients undergoing PDT (Figure 6A, 6B and supplementary Fig.2).

To further determine the functional changes in tumor cells induced by plasma-derived exosomes, invasive abilities of cancer cells co-incubated with exosomes harvested at different time points during PDT were investigated in Matrigel-based migration assays (Figure 6C and D). While control cells were minimally invasive, E-Cadherin loss and Vimentin upregulation resulted in a significant increase in both motility and invasiveness in cancer cells pretreated with t1 and t2 exosomes. In contrast, co-incubation with t3 and t4 exosomes did not enhance tumor cell invasiveness, remaining at the level of invasion mediated by exosomes from plasma of normal controls.

## DISCUSSION

Epithelial cells are characterized by the ability to establish close connections with neighboring cells. Malignant transformation of epithelial cells induces a change of their phenotype culminating in a loss of cell adhesion and the acquisition of an invasive and migratory phenotype [13]. As the cargo of exosomes derived from cultured tumor cells or from plasma of cancer patients has been shown to contain various EMT-relevant molecules [30], it has been suggested that tumor-derived exosomes (TEX) present in plasma of patients with cancer might play a role in the regulation EMT progression. In this study, we tested this hypothesis by contrasting the effects induced by TEX isolated from serially collected plasma specimens of HNSCC patients responding to PDT.

Various biological pathways are known to promote EMT, regulating expression levels of cytoskeletal molecules or activating the EMT-relevant transcription factors. Cadherins are adhesion proteins that regulate the balance between suppression and promotion of tumor cell invasion. E-Cadherin functions as a suppressor of invasion and is downregulated in various cancers including HNSCC in general [36, 37] and in the patients in this study evaluated prior to or immediately after receiving PDT. While the cohort of patients we evaluated was small, all the patients in our study cohort had highly aggressive, therapy-refractive tumors, where a mesenchymal profile is expected. In contrast to E-Cadherin, N-Cadherin promotes migration and invasion, is often upregulated in advanced cancers and its expression correlates with poor prognosis in HNSCC [37, 38]. Transcriptional factors such as Slug, Snail and Twist are known to be the downstream targets of signaling pathways operating in EMT in concert with suppression of the epithelial profile, including that of E-Cadherin [39, 40]. Moreover, an inverse relationship between Snail and E-cadherin has been noted to associate with patients' outcome in breast cancer and oral squamous cell carcinoma [41, 42].For these reasons, levels of expression of mRNAs coding for these molecules in recipient tumor cells co-incubated with TEX was of special interest.

Using exosomes isolated from plasma of the HNSCC patients undergoing PDT, all of whom experienced a clinical benefit after therapy, we demonstrated that these exosomes: (a) carried a cargo of molecules and genes that recapitulated the EMT progression/regression of the parental tumor occurring in response to PDT; and (b) effectively induced EMT progression/regression in recipient tumor cells following *ex vivo* co-incubation. The inverse effects mediated by t1/t2 vs t3/t4 plasma- derived exosomes obtained from the same patients' plasma convincingly illustrate the dual capability of these exosomes to either promote or reverse mesenchymalization in recipient tumor cells. This dual potential of exosomes to modulate EMT is clearly dependent on the cargo they carry, which presumably reflects the changing molecular/ genetic content of the parent tumor cells responding to PDT. Thus, exosomes, which in cancer patients are likely to be enriched in TEX, emerge as potential biomarkers of the tumor response to therapy. Further, these plasma- derived exosomes are biologically active and carry a cargo of molecules/genes that induce mRNA and protein alterations in recipient tumor cells. The phenotypic and functional alterations driven by plasma-derived exosomes can either promote or suppress EMT in recipient cells. In patients with active disease refractory to therapy, plasma- derived exosomes serve to promote EMT. However, in the HNSCC patients responding to PDT, plasma-derived exosomes suppressed EMT progression in recipient tumor cells. In this paradigm, exosomes, and especially TEX that phenotypically and functionally recapitulate parent tumor cells, appear to act as non-invasive predictors of response to PDT on the one hand and as conveyors of messages that can modulate the EMT program on recipient cells on the other.

We and others have previously shown that PDT influences patients' outcome by promoting anti-tumor immune responses [43, 44]. Regression of distant metastasis after PDT of primary lesions has also been reported [45]. In this study, we provide evidence that PDT can also modulate the TME in human cancers through the exosome-mediated reversion of the mesenchymal profile and reducing the aggressiveness and invasiveness of the cancer. The involvement of exosomes isolated from plasma of HNSCC patients treated with PDT in this process introduces a new and highly effective biological communication strategy that promises to be useful in future monitoring of responses of cancer patients to oncological therapies.

While this retrospective study has limitations due to a small patient numbers and the paucity of experiments investigating the mechanistic insights of TEX-mediated effects, its merit lies in the demonstration that serial monitoring of the exosome cargo in patients' plasma during the delivery of anti-cancer therapy informs about the therapy-induced changes in the tumor and hence about outcome.

## MATERIALS AND METHODS

### Study design

Nine HNSCC patients were treated with photodynamic therapy using Temoporfin (Foscan®, Biolitec, Austria) in a palliative setting due to recurrence of cancer between 2014 and 2016, as previously described [11]. Peripheral blood samples of all patients were collected after control of the patient's hemoglobin level at four different time points: 24 hours before PDT (t1), 24 hours after PDT (t2), one week after PDT (t3) and 4 to 6 weeks after PDT (t4). All patients had undergone various therapeutic treatments such as primary radio-chemotherapy and/or surgery according to the oncologic guidelines. PDT treatment was performed in general anesthesia, and the patients were hospitalized for a minimum of 7d with decreasing light restrictions. Table I provides information on the clinicopathological characteristics of these patients and additional information can be also found in ref. [11]. The follow-up period was from 5 to 112 months, with a mean of 43.6 months. Seven patients were cancer free and 2 patients still had a tumor, but the tumor load was decreased. The overall survival of all patients was 45 months.

Peripheral blood was also obtained from five healthy donors. Plasma samples were used for exosome isolation to serve as controls in experiments using patient-derived plasma exosomes. At least three biological replicates were performed for each experiment with the exact numbers specified in figure legends.

### Statistics

Analysis were conducted using GraphPad Prism 5 software. Comparison between continuous variables was performed using the 2-tailed paired t-test. P<0.05 was considered significant. Overall comparison between different time points was performed using one-way Anova.

### Collection of plasma

After isolation of PBMCs as described [11], plasma was centrifuged for 10 min at 2,500xg and collected for storage at −20°C. Plasma samples were thawed and batched, so that all samples of every patient were assayed together to avoid inter-measurement errors. Clinicopathological information for all patients can be found in our previous publication [11].

### Exosome isolation by mini size-exclusion chromatography (mini-SEC)

The mini-SEC method for exosome isolation was developed and optimized as previously described [46]. Briefly, plasma samples were thawed and centrifuged first at 2,000xg for 10 min at room temperature (RT) and then for 30min at 14,000xg at 4°C. Next, plasma was ultrafiltered using a 0.22μm filter (EMD Millipore, Billerica, MA, USA). An aliquot of plasma (1mL) was placed on a mini-SEC column and eluted with PBS. The void volume fractions #3 - #5 enriched in exosomes were collected (1mL/fraction). The fraction #4 contained the majority of isolated exosomes as previously reported [46] and was used for all exosome studies. The fraction #4 exosomes are referred to as “total exosomes.”

### BCA protein assay and sample concentration

The protein concentration in the fraction #4 was measured using the Pierce BCA protein assay kit (Pierce Biotechnology, Rockford, lL, USA) according to the manufacturer's instructions. The protein concentrations were calculated as μg protein/1mL plasma. Concentration of 1mL specimens was performed using Vivaspin 500 (VS0152, 300,000 MWCO, Sartorius, Göttingen, Germany).

### Exosome Characterization

#### Dynamic Light Scattering

Size measurements of exosomes were done using a Zetasizer (Malvern Instruments Ltd, England, UK). Exosomes diluted in PBS (1:100) were analyzed in equilibration time of 120 sec at the constant temperature of 25°C.

#### Nanotracking analysis

Exosomes were diluted to an appropriate level with particle-free PBS and continuously fed into the Nanoparticle Tracking Analysis (NTA) (NanoSight, Amesbury, UK) LM-10 system with a syringe pump. The Brownian motion of each individual exosomes within the field of view was visualized with a laser illumination unit and a high-definition CCD camera. Each measurement was recorded for 1 min and repeated for three times. The size distribution of exosomes was then analyzed and extracted from the motion of exosomes using the software that came with the NTA system.

### Transmission Electron Microscopy

Isolated total exosomes were fixed with 4% glutaraldehyde (Electron Microscopy Services, Hatfield, PA, USA) for 20min at RT. A 10μL droplet of glutaraldehyde- fixed exosomes was placed on Formvar-coated 300 mesh copper grid (Electron Microscopy Services, Hatfield, PA). The sample was incubated for 1min followed by rinsing with DI water for 1 min to ensure removal of PBS salts. Excess liquid was blotted-off with a Whatman filter. Post rinsing, 50 µl of Uranyl-acetate solution was put on the grid and allowed to remain for 1 min. Excess liquid was removed, and the grids were viewed on a Hitachi H-7100 transmission electron microscope (TEM, Hitachi High Technologies) operating at 100 keV. Digital images were collected using an AMT Advantage 10 CCD Camera System (Advanced Microscopy Techniques) and inspected using NIH ImageJ software.

### Western blots

Western blots for exosome proteins TSG101 and CD63 were performed as previously described [26].

### Detection of surface proteins on exosomes

For detecting exosome-associated surface proteins, on-bead flow cytometry was performed following exosome capture on streptavidin magnetic beads coated with biotin- labeled anti-CD63 mAbs. The detailed protocol was previously reported [47]. The following labeled detection Abs were used for staining: anti-E-cadherin (Biolegend, # 324104), anti-N-cadherin (Biolegend # 350808) or and anti-TGF-β (R&D, # FAB2463P). Immediately following 1h staining with Abs at RT and washing with buffer, flow cytometry was performed using a Gallios instrument. Samples were run for 2min and 10,000 events were acquired. For each detection Ab, corresponding isotype controls were included.

### Cell lines

For all experiments, the following squamous cell carcinoma lines were used: PCI13, a HNSCC HPV-negative cell line and SCC-90, a HNSCC HPV+ cell line were established and maintained in our laboratory [48]. The other cell lines used: A549 (human lung adenocarcinoma), MDAMB231 (human breast adenocarcinoma) and BT549 (human breast ductal carcinoma) were obtained from the ATCC and were provided to us by Dr. Peter Lucas (Children's hospital, Pittsburgh). All cell lines were routinely tested for Mycoplasma and were contamination free. Cells were cultured in 150cm2 cell culture flasks in 25ml of DMEM supplemented with α1% (v/v) penicillin and streptomycin and 10% (v/v) exosome-depleted fetal bovine serum (FBS, Gibco, Fisher Scientific, Pittsburgh, PA) at 37°C in an atmosphere of 5% CO2 in air. The cells were harvested upon reaching confluency of 60% to 80%.

### TaqMan analysis of mRNA expression levels in exosomes

To evaluate expression levels of EMT-related genes, TaqMan analysis was performed. Aliquots (20x104) of tumor cells were co-incubated with exosomes (10μg protein) for 96h and cells were then harvested using RLT buffer (Qiagen, Hilden, Germany). RNA was extracted using an RNeasy Kit (Qiagen). The RNA was used for cDNA synthesis and expression analysis by TaqMan in the StepOnePlus system (Applied Biosystems, CA, USA) was performed. Commercially-available TaqMan primers (Thermo Fisher Scientific Life Technologies, MA, USA) were purchased to evaluate local expression of the key mesenchymal and epithelial markers Vimentin, E-Cadherin, N-Cadherin, Slug, Snail, Twist, ZEB-1 and α-SMA (see Supplemental Table 1 for a list of all assay IDs). The expression of each gene was normalized to the HPRT1 housekeeping gene and calculated as fold increase [49].

**Table 1:** Clinicopathological data

	Patients (n=9)	
	n	%
*Age (years)*		
≤ 60	4	44
> 60	5	56
(range: 52-79)		
*Gender*		
male	6	67
female	3	33
*Tumor stage*		
pT1	3	33
pT2	0	0
pT3	1	11
pT4	5	56
*Nodal status*		
N0	3	33
N≥1	6	67
*Distant metastasis*		
M0	8	89
M1	1	11
*Amount of treatment lines (pre-PDT)*		
1	1	11
2	4	45
3	2	22
4	2	22
*Radiotherapy*		
0	1	11
1	5	56
2	3	33
*Chemotherapy*		
0	4	45
1	4	44
2	1	11
*HPV status*		
positive	1	11
negative	8	89

### Flow Cytometry of tumor cells

#### Detection of surface and intracellular proteins in cancer cells

Aliquots of cell lines, PCI13 and A549 (50x104 cells/ stain suspended in FACS buffer) were co-incubated with exosomes for 96h and were harvested by using cold PBS. For surface staining, non-specific binding was blocked by using a house-made blocking buffer (1% BSA, 1% human serum albumin (HSA) in PBS) for 15min. Incubation with the appropriate labeled Abs specific for Vimentin; Abcam #ab128507 and EpCAM; Abcam #ab187293) was performed for 1h. For intracellular staining, cells were first treated with FIX-Perm buffer (Biolegend, #421403) for 40min at 40C. After washing with Perm buffer and blocking of non-specific binding, staining with Vimentin conjugated Ab was performed for 30min at RT. Flow cytometry was performed using a Gallios instrument equipped with Kaluza 1.0 software (Beckman Coulter, Krefeld, Germany). Isotype controls were included in all flow cytometry experiments.

#### Immunofluorescence

Tumor cells (0.1 x 106) were plated on collagen type I-coated 18mm coverslips and allowed to adhere overnight. Next day, fresh medium containing 20μg/mL of exosomes was added. After 72h co-incubation, cells were washed in PBS, fixed in 3.33% PFA for 20min at RT and were permeabilized with 0.1% TritonX (Millipore-Sigma, St. Louis, MO, USA) for 10min. To minimize non-specific binding, blocking was performed with 10% goat serum for 20min at RT. Cells were then rinsed with wash buffer (PBS, 0.1%BSA) and incubated with 1:100 dilution (PBS with 0.1% BSA) of primary antibodies: mouse anti- human Vimentin (Ab8069; Abcam, Cambridge, MA) and rabbit anti-EpCAM (Ab71916; Abcam, Cambridge, MA) overnight at 4oC. Cells were rinsed 3x with wash buffer and incubated with 1:500 (PBS, 1% BSA) dilution of secondary antibodies: goat anti-mouse Dylight 488 nm (4408S, Cell Signaling Technologies, Danvers, MA, USA) and goat anti-rabbit Dylight 647 nm (4414S, Cell Signaling Technologies, Danvers, MA) for 2h at RT. Lastly, cells were rinsed 5x with wash buffer and imaged using a Zeiss LSM 880 confocal microscope (Carl Zeiss, Thornwood, NY, USA).

### Functional assays

#### Proliferation

Proliferation was quantified by using direct CyQUANT nucleic acid-sensitive fluorescence assay (Thermo Fisher Scientific, Waltham, MA, USA) according to the manufacturer's instructions. Briefly, 200 µL aliquots of cell suspension containing 1.25 × 103 cells/ mL were plated in wells of a 96-well microplate (Corning Inc., Corning, NY, USA) and allowed to adhere for 4h. Exosomes (10 µg/mL) were added to respective treatment wells and co-incubated with tumor cells for 72h. Next, cells were labeled with CyQUANT® Direct and fluorescence intensities were measured with TECAN spectrophotometer reader (#TECAN, Männedorf, Switzerland). Proliferation was assessed by plotting relative fluorescence intensities.

#### Spheroid assays

3D-spheroid assays were performed following the IncuCyte 96-well Kinetic 3D Spheroid Protocol (Essen BioScience, Ann Arbor, MI, USA). Cells (2.5x102/200 µL medium) were seeded in wells of Ultra Low attachment plates (Cat # ULA7007, Corning Inc, Corning, NY, USA) and cultured in DMEM media containing 5%FBS and 2% Cultrex BME (Trivigen, Gaithersburg, MD, USA) to allow spheroid formation. After 3 days, 100 µL of medium containing exosomes (10µg/mL) was added to each well and spheroid growth was monitored for 12 additional days, replacing the medium every 3 days. Spheroid growth was monitored by phase contrast images taken every 24h with a 10X objective. Spheroid volumes were calculated post 12 days using SpheroidSizer (MATLAB- based open-sourced software).

#### Migration assay

Migration assays were performed in in wells of 24 Transwell plates with an 8-µm pore-size polycarbonate filter (#ECM508; Millipore-Sigma, St. Louis, MO, USA). All media and supernatants were brought to room temperature before usage. The lower chamber contained 650 µL of DMEM complete media (10% FBS) supplemented with rhEGF (10ng/ml, AF-100-15, Peprotech, Rocky Hill, NJ, USA) and the upper chamber contained PCI13 or A549 cells (1 X 105 cells in 10 µL of medium with 2% FBS in media) pretreated with or not pretreated with exosomes for 96h. After an incubation period of 36h, the cells in the upper chamber (non-migrated cells) of the Transwell were removed, and the cells migrated to the lower side of the membrane were fixed with 4% glutaraldehyde for 10min and stained with 0.2% crystal violet for 10min at RT. Five random bright-field images were taken per Transwell membrane using a 5X objective of a Zeiss Axiovert 200M microscope (Carl Zeiss Microimaging, Thornwood, NY, USA). The number of migrated cells per condition was manually counted and subtracted from the number of cells migrated towards plain medium alone.

### Chemotaxis assays

Chemotaxis assays were performed using IncuCyte ClearView 96-well #137 plates (Essen BioScience) containing Transwell membranes (#143) with 8 µM diameter pores. Next, 2.5x103 tumor cells (MDAMB231 or BT549 cells) suspended in 6 µL of 0.5% FBS were placed in the upper chamber and allowed to adhere to the membrane surface for 30min at RT. Exosomes (2 µg protein) isolated from plasma of the HNSCC patients or normal donors were suspended in 200 µL of medium containing 1% (v/v) FBS and placed in lower chambers of the Transwell plate. Images of cells collecting at the upper and bottom surfaces of each ClearView membrane were obtained every 2h for 3 d using an IncuCyte live-cell Imaging System. The IncuCyte Chemotaxis Analysis software module was used to quantify migration of tumor cells towards medium containing plasma-derived exosomes.

### Invasion assays

Migration assays were performed in in wells of 24 Transwell plates with 8-um pore-size polycarbonate filter (#ECM508; Millipore-Sigma, St. Louis, MO). The 8-um pore-size polycarbonate filter was coated with 1 mg/ml Cultrex® RGF BME Type 2 (Trevigen, Gaithersburg, MD, USA). The lower chamber contained 650 µl of DMEM media supplemented with 0.1% FBS and 10 ng/ml rhEGF and the upper chamber had 1 X 105 cells (100 µL of 0.1% FBS media) of either PCI13, A549 or SCC90 cells pretreated with or without exosomes for 96h. After an incubation period of 36h, non-migrating cells in the top chamber Transwell membranes were removed and membranes were fixed and stained according to the Diff–Stain Kit staining protocol (Cat No: K7128, IMEB Inc., San Marcos, CA, USA). The quantification and analysis were performed as described above (Migration assays).

### Study approval

The acquisition of blood samples (patients and healthy controls) and clinical data for research purposes, was approved by the local ethics committee (# 323/14, University of Ulm, Germany). A written informed consent of all patients and healthy controls was received prior to inclusion in this study.

## SUPPLEMENTARY MATERIALS



## Abbreviations

αSM: Aalpha-smooth muscle actin
CTA: cancer testis antigens
EMT: epithelial-to-mesenchymal transition
EVs: extracellular vesicles
HNSCC: head and neck squamous cell carcinoma
PDT: photodynamic therapy
TEM: ransmission electron microscopy
TEX: tumor-derived exosomes
TME: tumor microenvironment
