# Supplementary material for: Plasma-derived Exosomes Reverse Epithelial-to-Mesenchymal Transition after Photodynamic Therapy of Patients with Head and Neck Cancer
Source: Oncoscience. 2018 Apr 29;5(3-4):75–87. doi: 10.18632/oncoscience.410 (PMC5978437; doi:10.18632/oncoscience.410)
Supplement: Supplementary file 1 [file oncoscience-05-0075-s001.pdf]

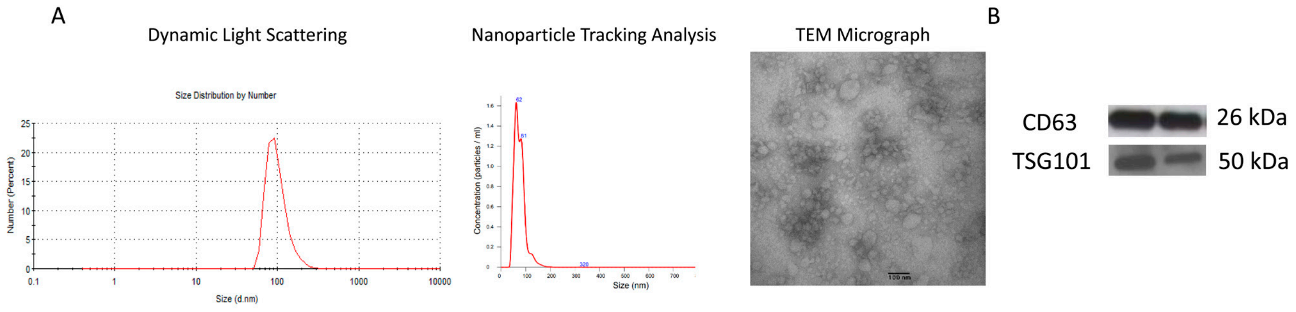

Supplementary Figure 1: (A). Characteristics of exosomes isolated by miniSEC from plasma of HNSCC patients treated with PDT. In B, Western blot of the isolated exosomes showing indicating the presence of CD63 and TSG101.

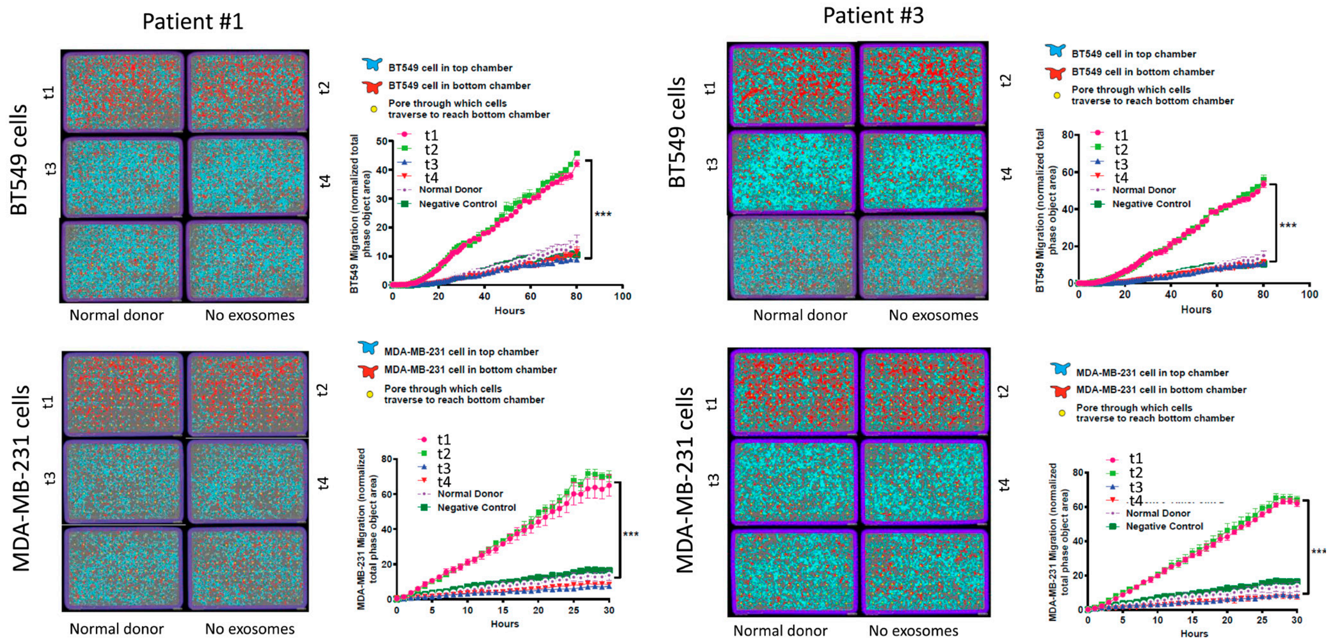

Supplementary Figure 2: Representative data for the chemotaxis assays using exosomes obtained from plasma of patients #1 and #3 as chemoattractants.

Supplementary Table 1: Primer IDs

Primer names	Assay ID
HPRT-1	Hs02800695_m1
α-SMA	Hs01081558_m1
Twist-For	Hs01675818_s1
E-cad-For	Hs01013958_m1
N-cad-For	Hs00354987_m1
Vim-For	Hs00185584_m1
Slug-For	Hs00161904_m1
Snail-For	Hs00195591_m1
ZEB1-For	Hs01566408_m1
